# Supplementary material for: Development and Evaluation of an Enzyme-Linked Immunosorbent Assay Targeting Rabies-Specific IgM and IgG in Human Sera
Source: Viruses. 2023 Mar 29;15(4):874. doi: 10.3390/v15040874 (PMC10142732; doi:10.3390/v15040874)
Supplement: Supplementary file 1 [file viruses-15-00874-s001.zip › viruses-2291153-supplementary.pdf]

Table S1. Results of the IgM IgG, Protein A ELISAs and RFFIT for all subjects at all timepoints tested. ELISA results are in EU/mL values and RFFIT in IU/mL values.

Subject #	Sample ID:	DPV:	IgM EU/mL	IgG EU/mL	Prot A EU/mL	RFFIT IU/mL
1	RAE-1	0	0.125	0.313	0.063	0.05
	RAE-2	14	14.39	3.42	3.30	17.5
	RAE-3	21	16.38	4.27	3.86	23.9
	RAE-4	28	6.69	19.14	13.84	106.8
	RAE-5	42	3.32	20.58	13.02	84.7
2	RAE-6	0	0.125	0.313	0.063	0.05
	RAE-7	7	1.64	0.313	0.063	2.0
	RAE-8	14	8.22	1.53	1.05	12.5
	RAE-9	21	5.73	2.06	1.56	11.3
	RAE-10	28	13.00	15.33	18.25	28.6
	RAE-11	42	4.68	18.37	18.07	27.3
3	RAE-12	0	0.125	0.313	0.063	0.05
	RAE-13	7	0.125	0.313	0.063	0.05
	RAE-14	10	0.38	0.313	0.063	0.2
	RAE-15	14	2.65	1.38	0.75	3.2
	RAE-16	21	5.22	4.85	8.97	11.9
	RAE-17	28	2.33	11.14	7.47	10.1
	RAE-18	42	1.25	18.57	27.57	24.4
4	RAE-19	0	0.125	0.313	0.063	0.05
	RAE-20	7	0.69	0.313	0.063	0.5
	RAE-21	14	7.04	1.91	1.40	9.9
	RAE-22	21	7.96	3.52	2.95	11.3
	RAE-23	28	3.29	15.38	16.62	26.1
	RAE-24	42	2.28	13.86	12.38	18.5
5	RAE-25	0	0.125	0.313	0.063	0.05
	RAE-26	7	1.17	0.313	0.063	0.9
	RAE-27	21	6.23	8.70	7.73	25.0
	RAE-28	28	2.90	8.48	6.42	13.5
6	RAE-29	0	0.125	0.313	0.063	0.05
	RAE-30	7	0.125	0.313	0.063	0.05
	RAE-31	14	1.23	0.27	0.24	0.6
	RAE-32	21	1.74	1.18	0.46	1.2
	RAE-33	28	1.14	0.313	0.55	1.2
	RAE-34	42	0.91	5.85	3.92	2.9
7	RAE-36	0	0.125	0.313	0.06	0.05
	RAE-37	16	8.03	2.381	1.46	10
	RAE-38	31	3.63	13.55	9.23	32.4
8	RAE-39	0	0.13	0.31	0.063	0.05
	RAE-40	16	0.125	0.313	0.063	0.05
	RAE-41	31	5.02	3.74	3.72	5.3
9	RAE-42	0	0.125	0.313	0.063	0.05
	RAE-43	7	0.36	0.313	0.063	0.05
	RAE-44	21	2.84	2.93	1.94	5.3
	RAE-45	42	1.04	9.46	9.62	14.3
10	RAE-55	0	0.125	0.313	0.063	0.05
	RAE-56	7	0.125	0.313	0.063	0.05
	RAE-57	14	2.23	8.86	6.92	13.1
	RAE-58	28	1.60	3.25	2.19	7.7

Note: results with values below the assay range (</=) are reported in chart as the assay lower limit/2.

Table S2. Average results (EU/ml or IU/mL values per assay) with + or - 2 standard deviation values by Day Post Vaccination (DPV).

**DPV**	**IgM EU/mL**	**IgG EU/mL**	**Prot A EU/mL**	**RFFIT IU/mL**
Day 7	1.37+/-5.02	0.5+/-1	0.1+/-0.18	0.24+/-0.68
Day 14	5.49+/-9.6	2.51+/-5.54	1.9+/-4.52	8.36+/-12.74
Day 21	6.59+/-9.6	3.93+/-4.9	3.93+/-6.46	12.84+/-17.66
Day 28	4.4+/-7.3	10.03+/-12.96	8.7+/-12.66	25.74+/-64.66
Day 42	2.24+/-3	14.45+/-11.64	14.09+/-16.12	28.68+/-57.5

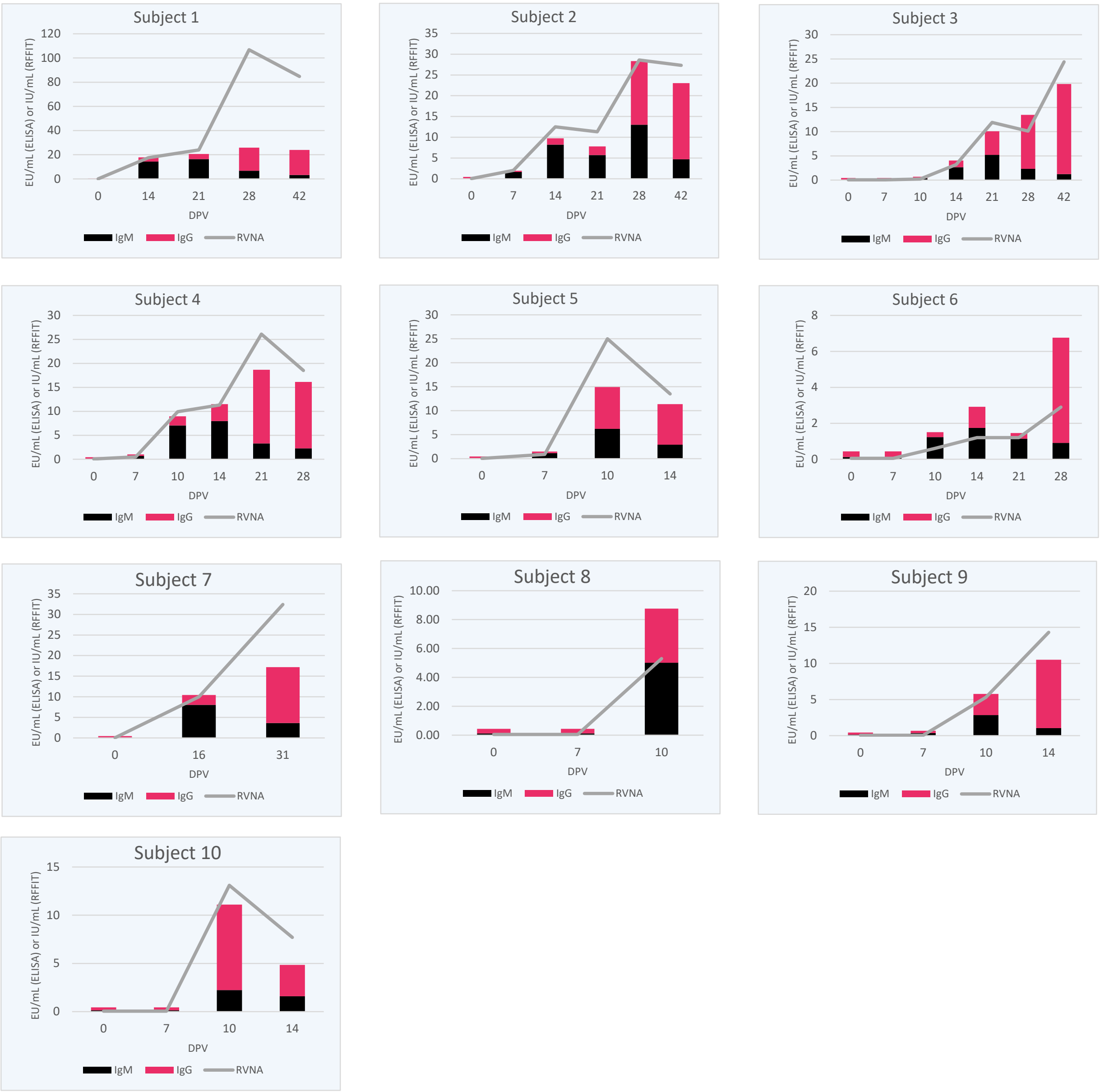

Figure S1. Individual variation in rabies antibody kinetics post-PrEP was demonstrated in three subjects, 1 in panel (a), 6 in panel (b), and 3 in panel (c). The IgM ELISA result is in black, and the IgG ELISA result is in magenta in the stacked bar, the RVNA (RFFIT) result is in the gray line.
